# A morpho-phylogenetic update on ixodid ticks infesting cattle and buffalos in Vietnam, with three new species to the fauna and a checklist of all species indigenous to the country

**DOI:** 10.1186/s13071-024-06384-5

**Published:** 2024-07-25

**Authors:** Sándor Hornok, Róbert Farkas, Ngoc Nhu Duong, Jenő Kontschán, Nóra Takács, Gergő Keve, Duan Ngoc Pham, Thanh Thi Ha Dao

**Affiliations:** 1 Department of Parasitology and Zoology, University of Veterinary Medicine, Budapest, Hungary; 2 Hungarian Research Network (HUN-REN) – University of Veterinary Medicine Budapest (UVMB) Climate Change: New Blood-Sucking Parasites and Vector-Borne Pathogens Research Group, Budapest, Hungary; 3 Department of Parasitology, National Institute of Veterinary Research, Hanoi, Vietnam; 4 Plant Protection Institute, Hungarian Research Network (HUN-REN) Centre for Agricultural Research, Budapest, Hungary; 5 Department of Plant Sciences, Albert Kázmér Faculty of Mosonmagyaróvár, Széchenyi István University, Mosonmagyaróvár, Hungary; 6 Department of Parasitology, Hanoi Medical University, Hanoi, Vietnam

**Keywords:** Ixodid ticks, *Rhipicephalus*, *Rhipicephalus microplus*, *Amblyomma*, *Haemaphysalis*, *cox*1, 16S rRNA

## Abstract

**Background:**

Southeast Asia is regarded as a hotspot for the diversity of ixodid ticks. In this geographical region, Vietnam extends through both temperate and tropical climate zones and therefore has a broad range of tick habitats. However, molecular-phylogenetic studies on ixodid tick species have not been reported from this country.

**Methods:**

In this study, 1788 ixodid ticks were collected from cattle, buffalos and a dog at 10 locations in three provinces of northern Vietnam. Tick species were identified morphologically, and representative specimens were molecularly analyzed based on the cytochrome *c* oxidase subunit I (*cox*1) and 16S rRNA genes. Fifty-nine tick species that are indigenous in Vietnam were also reviewed in the context of their typical hosts in the region.

**Results:**

Most ticks removed from cattle and buffalos were identified as *Rhipicephalus microplus*, including all developmental stages. Larvae and nymphs were found between January and July but adults until December. Further species identified from cattle were *Rhipicephalus linnaei*, *Rhipicephalus haemaphysaloides*, *Amblyomma integrum* and *Haemaphysalis cornigera*. Interestingly, the latter three species were represented only by adults, collected in one province: Son La. The dog was infested with nymphs and adults of *R. linnaei* in July. Phylogenetically, *R. microplus* from Vietnam belonged to clade A of this species, and *R. haemaphysaloides* clustered separately from ticks identified under this name in China, Taiwan and Pakistan. *Amblyomma integrum* from Vietnam belonged to the phylogenetic group of haplotypes of an *Amblyomma* sp. reported from Myanmar. The separate clustering of *H. cornigera* from *Haemaphysalis shimoga* received moderate support.

**Conclusions:**

Three tick species (*R. linnaei*, *A. integrum* and *H. cornigera*) are reported here for the first time in Vietnam, thus increasing the number of indigenous tick species to 62. Clade A of *R. microplus* and at least *R. linnaei* from the group of *Rhipicephalus sanguineus *sensu lato occur in the country. There is multiple phylogenetic evidence that different species might exist among the ticks that are reported under the name *R. haemaphysaloides* in South and East Asia. This is the first report of *A. integrum* in Southeastern Asia.

**Graphical Abstract:**

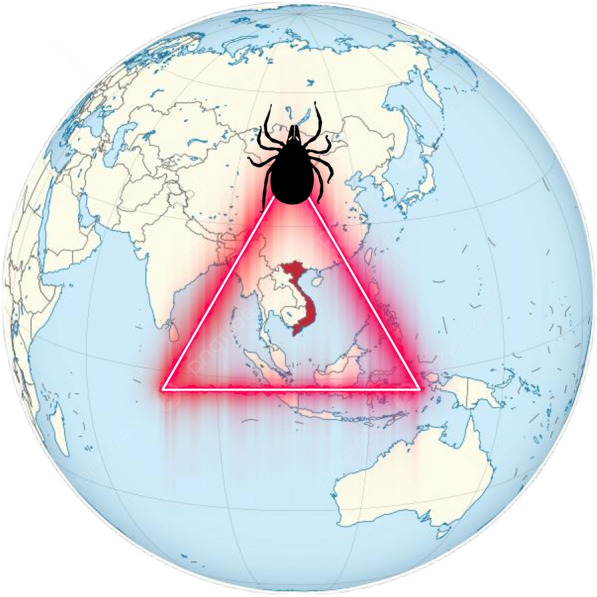

**Supplementary Information:**

The online version contains supplementary material available at 10.1186/s13071-024-06384-5.

## Background

Hard ticks (Acari: Ixodidae) have long been known for their high veterinary-medical importance, especially owing to the transmission of tick-borne pathogens which account for significant losses in terms of human and animal health and life. The global economic importance of ticks is particularly high for livestock, and tick-borne diseases especially affect regions with tropical climate [1]. Southeastern Asian countries (Myanmar, Thailand, Cambodia, Malaysia, Laos and Vietnam) belong to the Oriental Zoogeographic Region, characterized by having mainly tropical climate and consequently very rich species diversity [2]. For instance, compared to its land surface area, the Oriental Region probably has the highest number of insect species [3], and in Southeast Asia nearly twice as many spider species occur than in South Asia [4].

Knowledge on the tick fauna of continental Southeast Asia has intensified during the past decades [5]. This does not necessarily imply that the number of reported species will increase in time, because clarification of their taxonomic status may entail fewer well-established and recognized species in contrast to their ever-reported number (which may include taxonomically synonymous or questionable records). This is well exemplified by the fact that during the past 3 decades initially 96 hard tick species [6], then 100 [7] and finally 93 ixodid tick species were shown to be indigenous to Southeast Asia [5].

In some of the countries of this region, the number of tick species considered indigenous remained relatively constant until recently ([2, 5]: in Cambodia 17–18, in Malaysia 42–43, in Myanmar 34, in Thailand 58), with the exception of Laos where this number increased from 26 [5] to 30 [2]. Taken together, Southeast Asia should be regarded as a hotspot for the diversity of ixodid tick species, because this region represents only approximately 1.5% of the whole continental land surface but provides suitable habitats for the occurrence of at least 12.1% of all known tick species [2, 5, 6].

Importantly, in the whole of Southeast Asia, Vietnam was reported to have the highest number of ixodid tick species, i.e. 59 [5, 8-48] (Table 1). However, more recently only 57 were considered as unambiguously native to this country [2]. Vietnam has significant north-to-south expansion, overbridging the temperate and tropical climate zones. This bears impact on its faunal richness and species diversity, as illustrated by the highest number of *Haemaphysalis* species in a single geographical region [5]. Moreover, knowledge on the ixodid fauna of Vietnam bears high relevance to the potential transportation of a broad spectrum of tick species by birds in the direction of China and Japan to the north, as well as to Indonesia and even Australia in the south [49].

**Table 1:** Simplified review of the tick species and their hosts reported to occur in Vietnam

No	Tick species [reference on its occurrence in Vietnam]	Hosts of larvae and/or nymphs [reference]			Hosts of adults [reference]	
		In Vietnam	In Southeast Asia, in general		In Vietnam	In Southeast Asia, in general
1	*Ixodes acutitarsus* [5, 8, 9]	–	SM [10] (AV [11])		–	MM, LM (Bo), HU [10] (AV [11])
2	*Ixodes collaris* [12, 13]	Bats [12, 13]	–		Bats [12, 13]	–
3	*Ixodes granulatus* [14]	SM [14]	MA, HU, AV (RE) [5]		SM [14]	MA, HU, AV (RE) [5]
4	*Ixodes ovatus* [14–16]	–	SM, MM, HU [5, 11]		Civets [14]	MA, HU (AV) [5, 11]
5	*Ixodes simplex* [8, 16]	Bats [16]	Bats [8]		Bats [16]	Bats [8]
6	*Ixodes werneri* [16]	Rats, treeshrews [16]	SM [5]		Rats, treeshrews [16]	SM [5]
7	*Amblyomma crassipes* [14, 17–19]	Lizards, snakes [14, 17, 18]	RE (SM, MM) [5, 11]		RE (MA) [14, 17, 18])	RE (SM, MM) [5, 11]
8	*Amblyomma geoemydae* [14, 20, 21]	Turtles/tortoises [14, 22]	RE (MA, HU, AV) [5, 11]		Turtles/tortoises [14, 22]	RE (MA, HU) [5, 11]
9	*Amblyomma gervaisi* [9, 14]	Lizards, snakes [14]	RE (MA, AV) [5, 11]		Lizards, snakes [14]	RE (MA, AV) [5, 11]
10	*Amblyomma helvolum* [14, 20]	Lizards, snakes [14, 20]	RE (SM) [5]		Lizards, snakes [14, 20]	RE (LM) [5]
11	*Amblyomma javanense* [14]	Pangolin [14]	Pangolin (RE, MA, HU) [5, 21]		pangolin [14]	Pangolin (RE, MA, HU) [5, 21]
12	*Amblyomma pattoni* [14, 21]	Snakes (MA) [14]	Lizards, snakes (MA, AV) [5, 11]		Snakes (MA) [14]	Lizards, snakes (MA, AV) [5, 11]
13	*Amblyomma supinoi* [14, 21]	Turtles/tortoises [23]	RE (MA) [21]		Turtles/tortoises [14]	RE (MA) [21]
14	*Amblyomma testudinarium* [14]	MM, LM, (SM, HU) [14]	RE, MA, AV (frogs) [5, 11, 24]		Buffalos [14]	RE, LM: ungulates [5, 11]
15	*Amblyomma varanense* [14, 25]	Lizards, snakes [14]	RE, MM, LM [5, 11]		Lizards, snakes [14]	RE, MM, LM [5, 11]
16	*Dermacentor atrosignatus* [26]	–	SM, LM, HU [5]		Suidae [26]	RE, Suidae, MM, LM, HU [5]
17	*Dermacentor auratus* [14, 25]	SM [14]	MA (AV) [5, 27]		Suidae [14, 26]	Suidae, MM, LM, HU (RE) [5, 27]
18	*Dermacentor bellulus* [28]	Rats, treeshrews [28]	SM, MM (AV) [28]		HU [28]	Suidae, MM, LM (RE) [28]
19	*Dermacentor compactus* [14, 25, 29]	–	SM, MM (RE) [29]		Suidae, HU [14, 26]	Suidae, MM, LM (RE) [29]
20	*Dermacentor filippovae* [30]	–	–		Suidae [30]	–
21	*Dermacentor limbooliati* [31]	–	–		Suidae, HU [31]	–
22	*Dermacentor steini* [14]	–	SM [5, 32]		Suidae [26]	Suidae, MA, HU, RE [32]
23	*Dermacentor taiwanensis* [14, 28]	Rodents, dog [28]	SM, MM (AV) [28, 33]		–	Suidae, MM, LM [28, 33]
24	*Dermacentor tamokensis* [34]	–	–		Suidae [34]	Suidae [34]
25	*Haemaphysalis aborensis* [14]	Porcupine, badger [14, 18]	MM, AV [5, 35]		Ungulates [14, 18]	MM, LM, AV [5, 35]
26	*Haemaphysalis anomala* [14]	–	SM, AV [36]		Ungulates [14]	MM, Bo-LM, HU [36]
27	*Haemaphysalis asiatica* [14]	Carnivores [14]	SM, MM [37]		Carnivores [14]	Carnivores-Viverridae [37]
28	*Haemaphysalis atheruri* [14]	Bush-tailed porcupine [14]	Hystricidae [5]		Bush-tailed porcupine [14]	Hystricidae (SM, MM) [5]
29	*Haemaphysalis bandicota* [5, 16]	Rats [16]	SM (MM) [38]		Rats [16]	SM, MM (Bo) [38]
30	*Haemaphysalis canestrinii* [14]	–	Carnivores, SM, MM (AV) [39]		Dog [14]	carnivores, SM, MM (AV) [39]
31	*Haemaphysalis colasbelcouri* [14, 18]	–	–		Buffalos, HU [14, 18]	Bo, Cervidae [5, 40]
32	*Haemaphysalis dangi* [14, 18]	–	–		Porcupines, badgers, MM, ungulates [14, 18]	
33	*Haemaphysalis doenitzi* [14]	–	AV [5, 41]		Hare [14]	AV (hares, HU, RE) [5, 14, 41]
34	*Haemaphysalis formosensis* [14, 18]	Cervidae [14]	MA, AV [5]		Suidae [14]	MA, AV [5]
35	*Haemaphysalis grochovskajae* [14]	–	–		Bo [14]	–
36	*Haemaphysalis heinrichi* [14, 16]	Carnivores [14]	SM, carnivores [9, 11]		Carnivores [14]	Bo-MA, HU [9, 11]
37	*Haemaphysalis howletti* [14]	–	SM, MM, AV [5]		Hare [14]	SM, MM [5]
38	*Haemaphysalis hystricis* [14]	Dog [14]	Rodents, MM, LM, HU (AV) [5]		Dog [14], Suidae [26]	Dog, Bo, MM, LM, HU [42]
39	*Haemaphysalis koningsbergeri* [9]	–	–		–	SM, carnivores, ungulates, HU [5, 14]
40	*Haemaphysalis lagrangei* [14]	Carnivores, ungulates [14]	Carnivores, ungulates [43]		Carnivores, ungulates [14]	+ Rodents, primates-HU, AV [43]
41	*Haemaphysalis laocayensis* [9, 14, 18]	–	–		Otter, deer [14, 18]	–
42	*Haemaphysalis mageshimaensis* [14]	–	SM, AV [44]		Carnivores, ungulates [14]	MM, LM, HU [44]
43	*Haemaphysalis obesa* [14]	–	Carnivores, HU [5, 45]		Buffalo [14]	Ungulates, Ursidae, primates [45]
44	*Haemaphysalis ornithophila* [14]	–	AV [18]		Buffalo [14]	Hares, Mustelidae, Bo, AV [11, 18]
45	*Haemaphysalis papuana* [14]	–	MA [5, 46]		Dog [14]	Suidae-MA, HU (AV) [5, 14, 46]
46	*Haemaphysalis quadriaculeata* [14]	–	–		Dogs, SM, MM, HU [14, 16]	
47	*Haemaphysalis roubaudi* [14]	–	–		Deer, HU [14, 47]	
48	*Haemaphysalis shimoga* [14]	Rodents [14]	Rodents [11]		Large ungulates, HU [14, 18]	Rodents, ungulates, HU [11]
49	*Haemaphysalis spinigera* [14]	*Garrulax leucolophus* [14]	SM, MM, AV [14]		Dog, ungulates [14]	SM, carnivores, ungulates, HU [5, 11]
50	*Haemaphysalis suntzovi* [14]	–	–		Suidae, porcupine [14]	
51	*Haemaphysalis traguli* [14]	Mouse deer [26]	Trangulidae [5]		Mouse deer [14, 20]	Muridae, Suidae, Trangulidae [5]
52	*Haemaphysalis traubi* [14]	–	–		Deer [14]	MM, LM [5]
53	*Haemaphysalis wellingtoni* [14]	Shrews, carnivores, ungulates, primates-HU, AV [5, 14, 18]				
54	*Haemaphysalis yeni* [14]	Deer [14]	Hares, carnivores [11]		Deer [14]	Hares, carnivores, large ungulates [11]
55	*Hyalomma isaaci*	–	Hares, MM, AV [5, 11]		Cattle [14, 16]	MM, LM-Bo, HU [5, 11]
56	*Nosomma monstrosum*	–	SM [5, 11, 21]		Buffalo [14]	MM, LM-Bo (HU) [5, 11, 21]
57	*Rhipicephalus haemaphysaloides*	SM [14], dog, rat [48]	MA (AV) [5, 11]		Bo-LM [14], dog, rat [48]	Dog, Bo-MA, HU [5, 11]
58	*Rhipicephalus sanguineus s.l*	Dog [14, 18], cattle [48]	Carnivores (RE) [5, 11]		Dog [14, 18, 26], cattle [48]	Carnivores, HU [5, 11]
59	*Rhipicephalus microplus*	Bo, deer, carnivores [14, 48]	Bo, HU (AV, RE, frogs) [5, 11]		Bo, deer, carnivores [14, 48]	Bo, HU (AV, RE, frogs) [5, 11]

Human or rare hosts are indicated with underlined fonts or parentheses, respectively

MA: mammals; SM: small size mammals; MM: medium size mammals; LM: large size mammals; HU: humans; Bo: Bovidae; AV: birds; RE: reptiles

However, in Vietnam the latest large-scale tick surveys were conducted decades ago [14, 16, 18]. In addition, prior to the era of molecular methods, data of these studies were all based on morphological identification of tick species. As recently reported, the lack of reference sequences and standard taxonomic keys specific to native tick species makes morphological identification of Vietnamese ticks difficult [25]. Based on National Center for Biotechnology Information (NCBI) Nucleotide database, among *Ixodes* species only bat-associated ones were barcoded from Vietnam [12, 50]. Although several new *Dermacentor* species were described from specimens collected in this country [e.g. 28, 34], corresponding genetic data are not accessible. Considering other ixodid genera, only a single sequence of *Rhipicephalus sanguineus *sensu lato is available from Vietnam in GenBank [51].

In light of the above, the aims of this study were: (i) to collect large numbers of ticks from cattle and buffalo in northern Vietnam, (ii) to identify all specimens morphologically and (iii) to select representatives of each species for molecular-phylogenetic analyses based on two mitochondrial markers. In addition, it was also highly relevant in this context (iv) to review and update the list of all ixodid species already known or discovered here to occur in Vietnam. At the same time, this study is also meant as an initiative for barcoding all tick species of Vietnam.

## Methods

### Sample collection and morphological identification of tick species

Hard ticks were collected from 60 cattle, five buffalos and a dog between July 2022 and April 2023 at 10 locations in three provinces of Northern Vietnam (Table 2, Supplementary Table 1). None of the animals included in this study were imported from abroad. Depending on sampling conditions (e.g. restraint of animals and accessibility to affected skin surfaces), the great majority or all ticks were removed, allowing the estimation of mean infestation intensity: number of conspecific ticks divided by the number of hosts. All ticks were stored in 96% ethanol. Tick species were morphologically identified according to standard keys and illustrations [52–57]. Ticks were examined and pictures were made with a VHX-5000 digital microscope (Keyence Co., Osaka, Japan).

**Table 2:** Data of tick samples collected in Vietnam, listed according to tick species

Year	Data of collection				*Rhipicephalus microplus*					*Rh. linnaei*			*Rh. haema-physaloides*	*Amblyomma integrum*	*Haemaphysalis cornigera*
	Province	Host	*n* =	Month	Larva	Nymph	Male	Female	Infestation intensity	Nymph	Male	Female	Female	Female	Female
2022	Ha Noi	Cattle	11	July	–	7	16	132	14.1	–	1*	–	–	–	–
		Cattle	2	Sept	–	–	1	6	3.5	–	–	–	–	–	–
		Cattle	1	Dec	–	–	–	9	9	–	–	–	–	–	–
	Son La	Cattle	1	July	–	–	–	33	33	–	–	–	–	–	2*
	Thai Nguyen	Dog	1	July	–	–	–	–	–	3	43**	25**	–	–	–
		Cattle	1	July	2	3	34	114	153	–	–	–	–	–	–
		Cattle	3	Dec	–	–	9	45	18	–	–	–	–	–	–
2023	Son La	Cattle	11	Jan	–	5	15*	104*	11.3	–	–	–	–	–	–
		Cattle	3	Apr	–	–	–	–	–	–	–	–	2**	1*	–
	Ha Noi	Cattle	24	Apr	5	80	192**	553	34.6	–	–	–	–	–	–
		Buffalo	1	Apr	–	–	–	2	2	–	–	–	–	–	–
	Thai Nguyen	Cattle	2	March	3	245	2	7	128.5	1	–	–	–	–	–
		Cattle	1	Apr	–	1	7	5	13	–	–	–	–	–	–
		Buffalo	4	Apr	–	–	–	73	18.3	–	–	–	–	–	–
	In total from all hosts		66		10	341	276	1083	–	4	44	25	2	1	2

Infestation intensity was only estimated for *Rhipicephalus microplus*

^*^Molecularly also identified (the number of asterisks indicates the number of specimens analyzed with PCR and sequencing)

### DNA extraction and PCR analyses

Ticks were disinfected on their surface with sequential washing in 10% sodium-hypochlorite, tap water and distilled water. DNA was extracted with the QIAamp DNA Mini Kit (QIAGEN, Hilden, Germany), including an overnight digestion in tissue lysis buffer and Proteinase K at 56 °C. An extraction control (tissue lysis buffer) was also processed with the tick samples to monitor cross-contamination.

PCR amplification of an approximately 710-bp-long part of the cytochrome *c* oxidase subunit I (*cox*1) gene was performed with the primers LCO1490 (forward: 5ʹ-GGT CAA CAA ATC ATA AAG ATA TTG G-3ʹ) and HCO2198 (reverse: 5ʹ-TAA ACT TCA GGG TGA CCA AAA AAT CA-3ʹ), which are most widely used for barcoding ticks [58, 59]. The reaction mixture, in a volume of 25 µl, contained 1 U (0.2 µl) HotStarTaq Plus DNA polymerase, 2.5 µl 10 × CoralLoad Reaction buffer (including 15 mM MgCl_2_), 0.5 µl PCR nucleotide Mix (0.2 mM each), 0.5 µl (1 µM final concentration) of each primer, 15.8 µl ddH_2_O and 5 µl template DNA. The PCR was performed with the following conditions: an initial denaturation step at 95 ℃ for 5 min was followed by 40 cycles of denaturation at 94 ℃ for 40 s, annealing at 48 ℃ for 1 min and extension at 72 ℃ for 1 min. Final extension was performed at 72 ℃ for 10 min.

Another PCR was used to amplify an approximately 460-bp-fragment of the 16S rDNA gene of Ixodidae [60], with the primers 16S + 1 (5ʹ-CTG CTC AAT GAT TTT TTA AAT TGC TGT GG-3ʹ) and 16S-1 (5ʹ-CCG GTC TGA ACT CAG ATC AAG T-3ʹ). In the latter reaction, components and cycling conditions were the same as above, except for annealing at 51 °C.

### Phylogenetic analyses

In all PCRs, non-template reaction mixture served as negative control. Extraction controls and negative controls remained PCR negative in all tests. Purification and sequencing of the PCR products were done by Eurofins Biomi Ltd. (Gödöllő, Hungary). Quality control and trimming of sequences were performed with the BioEdit program. Obtained sequences were compared to GenBank data by the nucleotide BLASTN program (https://blast.ncbi.nlm.nih.gov). New sequences were submitted to GenBank under the following accession numbers (cytochrome *c* oxidase subunit I [*cox*1] gene: PP197235-PP197241, 16S rRNA gene: PP197249-PP197254). Sequences from other studies (retrieved from GenBank) included in the phylogenetic analyses had 99–100% coverage with sequences from this study. Sequence datasets were resampled 1000 times to generate bootstrap values. Phylogenetic analyses of *cox*1 and 16S rRNA sequences were performed with the Maximum Likelihood method, General Time Reversable (GTR) or Tamura-Nei models, respectively, according to the selection of the MEGA software [61, 62].

## Results

### Morphological identification, host-associations and spatiotemporal distribution of tick species

Altogether, 1788 ixodid ticks were collected. These belonged to five species (Table 2). Most (*n* = 1710) of ticks removed from cattle and buffalos were identified as *Rhipicephalus microplus*, including larvae (*n* = 10), nymphs (*n* = 341) and adults (*n* = 1359). Larvae and nymphs were found between January and July, while males and females were found until December. The estimated intensity of infestation ranged from a few ticks (December to April) up to 128 or 153 ticks per cattle in March and July, respectively (Table 2).

**Fig. 1:**
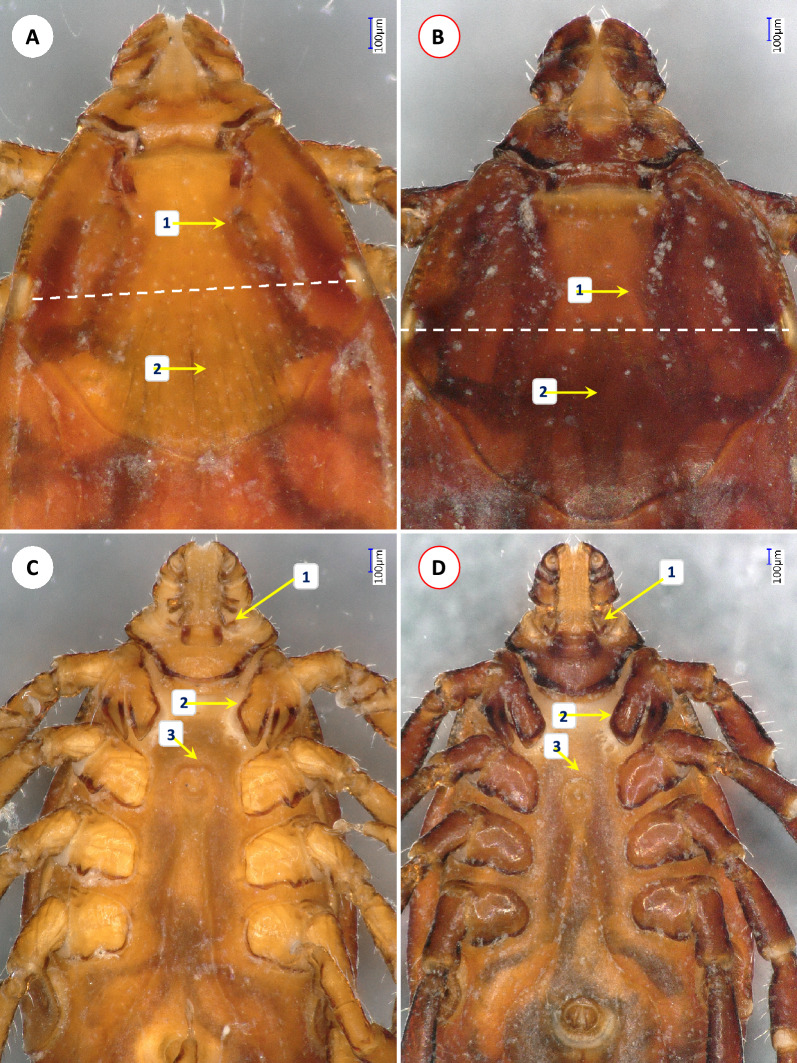
Morphological characters of *Rhipicephalus linnaei* (**A**, **C**) and *R. haemaphysaloides* (**B**, **D**) females collected in Vietnam. **A**, **B** Scutum and dorsal view of palps: (1) bending of cervical grooves **A** much anteriorly to or **B** near the level of eyes (white dashed line); (2) punctuations of caudo-central area of scutum **A** dense or **B** scarce. **C**, **D** Ventral view of basis capituli and coxae: (1) posteroventral palpal spur has **C** perpendicular or **D** acute angle; (2) medial edge of coxa I encloses **C** acute angle or **D** is parallel with the incision of coxa I; (3) genital groove anteriorly **C** flattened or **D** rounded.

**Fig. 2:**
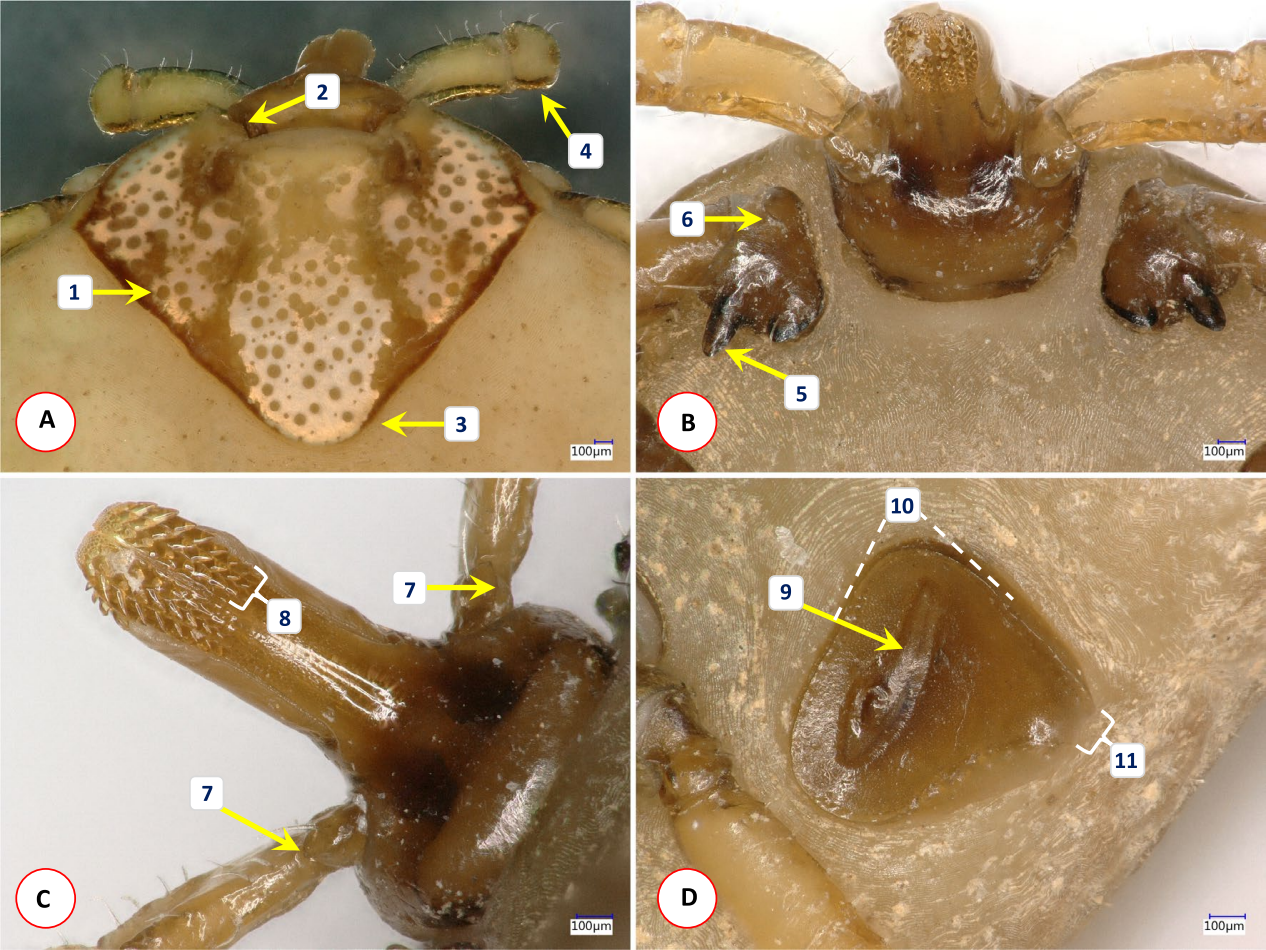
Morphological characters of *Amblyomma integrum* female collected in Vietnam. (A) Scutum and dorsal view of palps: (1) punctuations deep and large laterally, along the median line interspersed with shallow and small ones; (2) medial edge of scapular region slightly convex; (3) posterior margin narrow, rounded; (4) palpal segment III has small lateral and medial protuberance; (B) ventral view of basis capituli and coxae I: (5) the external spur of coxa I is much longer than the internal spur; (6) spur-like callosity anteriorly on coxa I; (C) ventral view of palpal basis and hypostome: (7) palpal segment I with a longitudinal, sharp ridge ending with a small, rounded spur; (8) dental formula 3/3; (D) spiracular plate: (9) opening surrounded by an elongate deepening approximately 2/3 of the length of plate; (10) medial and lateral margins enclose an acute angle; (11) dorsal prolongation short, narrow

**Fig. 3:**
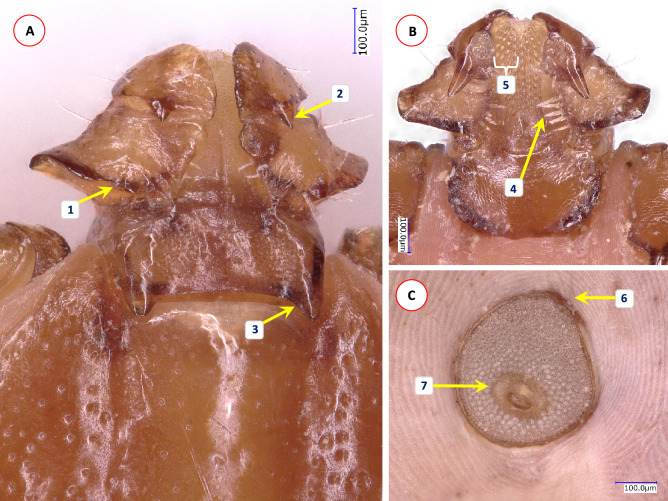
Morphological characters of *Haemaphysalis cornigera* female collected in Vietnam. (A) Dorsal view of basis capituli and palps: (1) posterior margin of palpal segment II wavy, with a prominent medial spur-like protrusion; (2) posterior margin of palpal segment III with sharp, triangular spur; (3) cornuae conspicuous, caudally directed, pointed; (B) ventral view of basis capituli and palps: (4) there are five infrainternal setae; (5) between the long, triangular spurs on palpal segments III the hypostome has a dental formula of 4/4; (C) spiracular plate subcircular or subquadrate, (6) dorsally with straight edge and hardly visible, blunt dorsal prolongation; (7) opening subcentral, surrounded with an oval area void of aeropyles

Four additional species identified among ticks from cattle were *Rhipicephalus linnaei* (*n* = 2; Fig. 1A, C), *Rhipicephalus haemaphysaloides* (*n* = 2; Fig. 1B, D), *Amblyomma integrum* (*n* = 1) (Fig. 2A–D) and *Haemaphysalis cornigera* (*n* = 2) (Fig. 3A–C). Interestingly, the latter three species were only represented by females, collected in one province: Son La (Table 2). The single dog sampled in this study was infested by nymphs and adults of *R. linnaei* (*n* = 71) in July (Table 2).

### Molecular-phylogenetic analyses of tick species

**Fig. 4:**
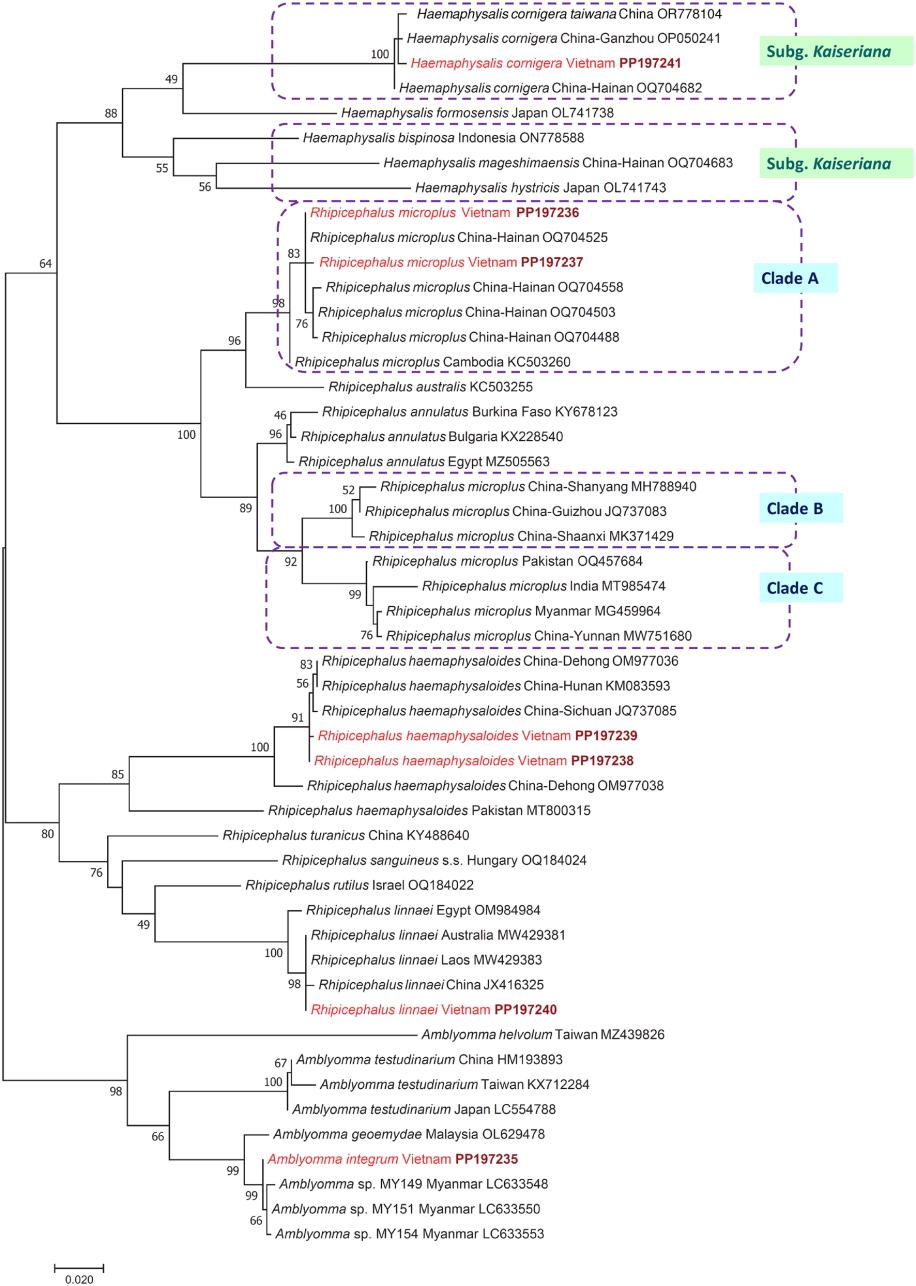
Phylogenetic tree of Metastriata based on the *cox*1 gene. Genera and species were selected based on geographical and taxonomical relevance to this study. In each row of individual sequences, the country of origin and the GenBank accession number are shown after the species name. Sequences from this study are indicated with red fonts and bold maroon accession numbers. The evolutionary history was inferred by using the Maximum Likelihood method and the General Time Reversible model. Sequence dataset was resampled 1000 times to generate bootstrap values. The tree is drawn to scale, with branch lengths measured in the number of substitutions per site. The analysis involved 50 nucleotide sequences, and there were a total of 633 positions in the final dataset

**Fig. 5:**
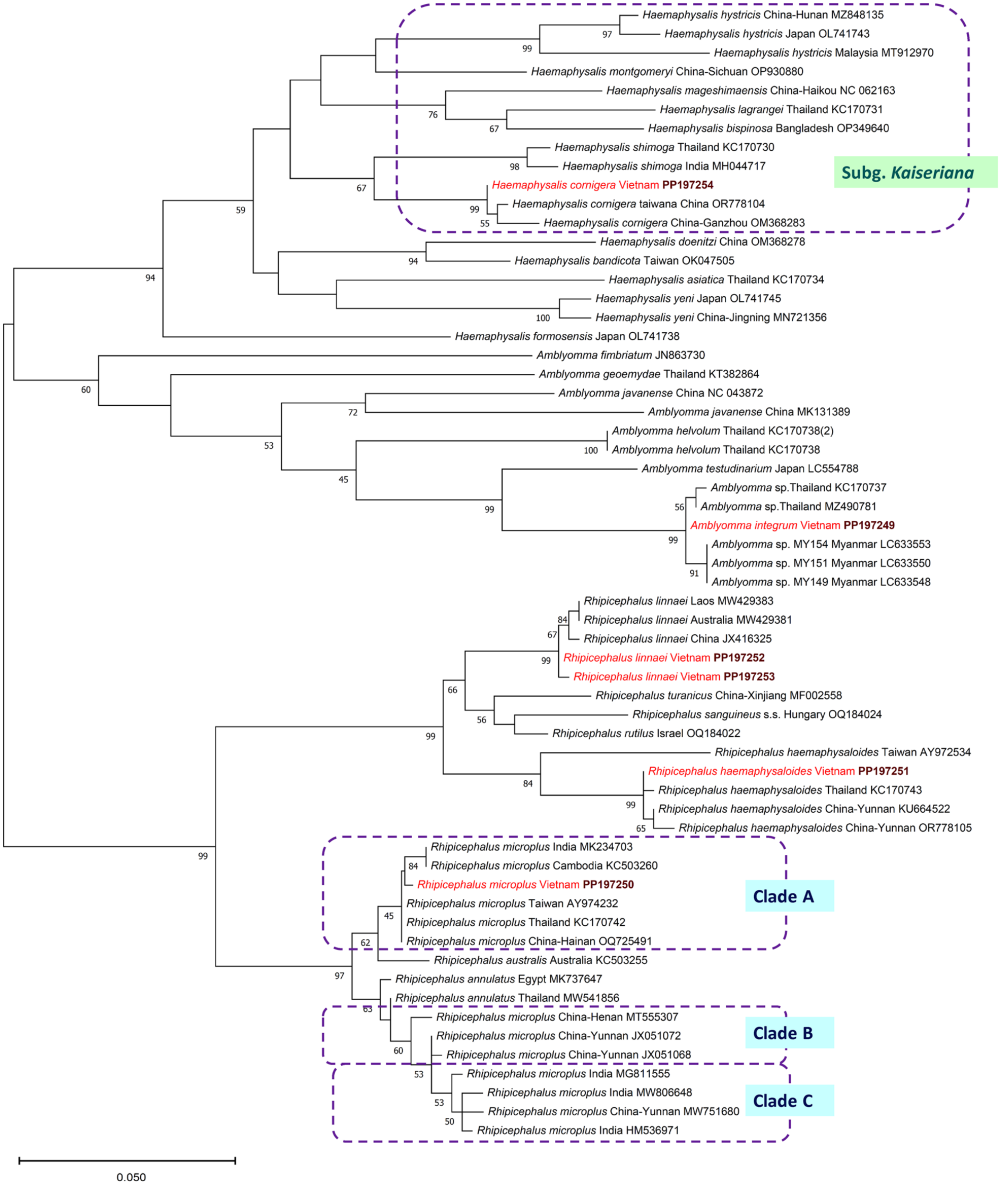
Phylogenetic tree of Metastriata based on the 16S rRNA gene. Genera and species were selected based on geographical and taxonomical relevance to this study. In each row of individual sequences, the country of origin and the GenBank accession number are shown after the species name. Sequences from this study are indicated with red fonts and bold maroon accession numbers. The evolutionary history was inferred by using the Maximum Likelihood method and the Tamura-Nei model. Sequence dataset was resampled 1000 times to generate bootstrap values. The tree is drawn to scale, with branch lengths measured in the number of substitutions per site. The analysis involved 60 nucleotide sequences, and there were a total of 454 positions in the final dataset

Among the four specimens of *R. microplus* that were molecularly analyzed, two *cox*1 haplotypes were identified. One of these (PP197236) had 100% (633/633 bp) identity in its *cox*1 sequence with several GenBank entries, among the others from the southernmost province of China (Hainan: OQ704525), Kenya (MT430985), South Africa (KY457541) and Colombia (MF363057). An isolate from Cambodia had lower (99.3%; 632/636 bp) sequence identity than this *R. microplus* isolate from Vietnam. Similarly, the 16S rRNA sequence of *R. microplus* from Vietnam (PP197250) was 99.8% (410/411 bp) identical to haplotypes reported from Thailand (KC170742), China (Hainan: OQ725491) and Taiwan (AY974232) but only 99.3% (407/410 bp) homologous to an isolate from Cambodia (KC503260). The Vietnamese haplotypes belonged to clade A of *R. microplus* as a sister group to *Rhipicephalus australis* (Figs. 4, 5).

The *cox*1 sequence of *R. linnaei* from Vietnam (PP197240) showed 100% identity to sequences of conspecific ticks from Laos (MW429383) and Australia (MW429381) and was also nearly identical (99.7%: 637/639 bp) to another from China (JX416325). The predominant 16 rRNA haplotype of *R. linnaei* from Vietnam (i.e. relevant to four out of five examined ticks) (PP197252) had (421/423 bp) identity to the corresponding sequence of ticks from Laos (MW429383) and China (JX416325). Both the *cox*1 and 16S rRNA sequences of *R. linnaei* from Vietnam clustered as a sister group to haplotypes including *Rhipicephalus rutilus* (Figs. 4, 5).

The two *cox*1 haplotypes of *R. haemaphysaloides* from Vietnam differed only in a single nucleotide: one of them (PP197238) was 99.7% (638/640 bp) identical to conspecific haplotypes available in GenBank from southern (Hunan: KM083593) and southcentral (Sichuan: JQ737085) provinces of China. Interestingly, *R. haemaphysaloides* from Vietnam was phylogenetically well separated from specimens reported under this species name from Southern China (Dehong: OM977038) and Pakistan (MT800315) (Fig. 4). Both representatives of this species identified in Vietnam had identical 16S rRNA haplotypes (PP197251) and had the closest sequence homology (99.8%; 420/421 bp) to *R. haemaphysaloides* from China (Yunnan: KU664522) and Thailand (KC170743) but only 94.3% (397/421 bp) to another haplotype from Taiwan (AY972534). These relationships were well reflected by the 16S rRNA phylogenetic analysis, indicating divergence within *R. haemaphysaloides* with high (84–85%) support (Figs. 4, 5). In addition, the sequence length coverage was lower (96%) and the identity 94.6% (383/405 bp) to a haplotype reported from Pakistan (MT799956).

The *cox*1 sequence of *A. integrum* (PP197235) had 99.1% (574/579 or 560/565 bp) sequence identity to those of conspecific ticks reported from India (OP473983, OQ318206, OQ306569), which were omitted from the phylogenetic analysis because of the low sequence length coverage. The identity was 99.7–99.8% (635–636/637 bp) to further specimens from Myanmar (LC633550, LC633553) not identified to the species level. On the other hand, *A. integrum* collected in Vietnam had only 98.3% (626/637 bp) *cox*1 sequence identity to *Amblyomma geoemydae* from Malaysia (isolate SGL03d: OL629478). The phylogenetic clustering (sister position) of *A. integrum* (Vietnam) and *A. geoemydae* (Malaysia) was well supported (Fig. 4), but their p-distance was low, 1.7% (11/637 bp). The 16S rRNA haplotype of *A. integrum* identified in Vietnam (PP197249) showed 99.5–99.8% (413/415 or 410/411 bp) sequence homology to isolates reported from Thailand (KC170737, MZ490781), a similar 99.5% (412/414 bp) to ticks morphologically not identified to the species level from Myanmar (e.g. LC633553) but only 93.3% (389/417 bp) identity to *Amblyomma testudinarium* reported from Japan (LC554788) and 87.4% (368/421 bp) to *A. geoemydae* from Thailand (KT382864). Phylogenetically, the latter two haplotypes clustered separately from the group of *A. integrum* collected in Vietnam (Figs. 4, 5). Importantly, the level of 16S rRNA sequence identity was very low (86.1%; 346/402 bp) and the p-distance high (13.9%) between the same isolate (SGL03d) of *A. geoemydae* from Malaysia (OL616095), which was compared above in the context of *cox*1 gene sequences.

The only *Haemaphysalis* species identified in this study, *H. cornigera* (PP197241), showed 99.5% (636/639 bp) *cox*1 sequence homology to specimens reported under this name in the southernmost province of China (Hainan: OQ704682) and Southeastern China (Ganzhou: OP050241). However, in the absence of *cox*1 sequence from the most closely related species, *Haemaphysalis shimoga* in GenBank, the nearest phylogenetic relationships of *H. cornigera* from Vietnam within its species group could not be evaluated based on this genetic marker. Importantly, the 16S rRNA haplotype of *H. cornigera* from Vietnam (PP197254) was only 94.6–94.8% (402/424–425 bp) identical to the corresponding sequence of *H. shimoga* reported from Thailand (KC170730) and India (MH044717), and their separate clustering received moderate (67%) bootstrap support (Fig. 5).

## Discussion

This study aimed at updating our knowledge on the tick infestation of cattle and buffalos in Vietnam, at the same time initiating the barcoding of ixodid species in this tick diversity hotspot. Previously, 59 species of ixodid ticks were reported to occur in Vietnam (Table 1). The present findings provide morphological and molecular evidence on the occurrence of three more species, which are thus newly recognized as indigenous to the fauna of the country.

From the list of 59 hitherto found ixodid species (Table 1), three species were excluded based of uncertainties in their indigenous or taxonomic status connected to Vietnam. First, *Rhipicephalus annulatus* was reported on cattle (probably introduced in this way) in Vietnam [63]. However, although the climatic conditions in Vietnam are suitable for its establishment [64], it is not regarded as indigenous [5]. On the other hand, *R. annulatus* was reported in Southeastern China close to Vietnam [65]; therefore, re-examination of formerly collected material and future monitoring of this ixodid species will be necessary to evaluate its presence in the fauna of Vietnam.

Second, *Africaniella* (formerly *Aponomma*) *orlovi* [66] was originally described from female ticks collected from Burmese python in Vietnam [67], unlike its sister species, *Af. transversale* reported from ball python in Africa and the Middle East. This species was also reported later in a confirmation of its indigenous status [14] but eventually excluded from the list of native ticks [16]. In the latter study, *Af. orlovi* was thought to originate from erroneously labeled specimens [16], but later it was still considered (at least provisionally) valid [11, 66]. Therefore, it is an important future task to try to access its specimens from larger snakes in northern Vietnam where the type specimen originated.

Third, *Ixodes pilosus* was also mentioned to occur in the country [18], but later this was rejected [16]. Nevertheless, in a later project the finding of *I. pilosus* in Central Vietnam was reported again [68].

The only tick species collected in this study, which provided sufficient numbers of developmental stages and data encompassing several months to evaluate its seasonality, was *R. microplus*. This species is regarded as the economically most important tick infesting cattle in a worldwide context [69, 70]. Notably, although nowadays it has a global geographical range in the tropics and subtropics, it is thought to originate in Southeast Asia, i.e. the region of Vietnam [71]. *Rhipicephalus microplus* belongs to the subgenus *Boophilus*, members of which have a one-host life cycle that can be completed in as short as 3–4 weeks and will typically result in heavy tick burdens [54], as also demonstrated in the present study. Its seasonality in Vietnam appears to be in line with previous studies in the region [72], with peak numbers in the summer. However, the small sample size does not allow to reach final conclusions about the complete seasonal activity of *R. microplus* in the study region.

*Rhipicephalus microplus* was genotyped in countries surrounding Vietnam, including Malaysia [73], Cambodia [74], Thailand [75], Myanmar, China [76] and Laos [55]. However, prior to this study, no sequences of *R. microplus* were available in GenBank from Vietnam, despite successful amplification efforts [25]. According to the results of this study, at least clade A of *R. microplus* occurs in this country, which is also the predominant genetic lineage in Southeast Asia [73, 76].

Based on literature data, the most important hosts of *R. microplus* are domestic and wild ungulates, as observed here, but occasionally also carnivores, rodents and even humans (Table 1; [48]). By contrast, the predominant hosts of *R. linnaei* are dogs [57, 77]. Nevertheless, in the present study, two specimens of this species were also collected from cattle (Table 2). Haplotypes of *R. linnaei* were reported from countries neighboring Vietnam (e.g. Laos and China: [77]) but discounting one sequence [51] reported as *R. sanguineus *s.l. from Vietnam, no simultaneous morphological and molecular evidence existed from this country on the occurrence of this species. Therefore, this is the first report on *R. linnaei*, identified as such in Vietnam.

A third species of the genus *R. haemaphysaloides* was also collected on one occasion from cattle in this study (Table 2). Typical hosts of this species include cattle as well as companion animals, wild animals and rodents in this geographical region [48]. In Vietnam, *R. haemaphysaloides* was reported from dogs and rats [48], as well as cattle [14], the latter in line with the present findings. Importantly, phylogenetic analyses of this study as well as of previous reports on this species focusing on Southern or Southeastern Asia [65, 78] reflect well-supported divergence, i.e. sister group relationship between haplotypes from Vietnam, different parts of China and Pakistan. These results suggest the existence of several cryptic or novel species in this taxon (group).

Although the genus *Ixodes* has the greatest number of species worldwide, only 14 are present in continental Southeast Asia [5]. However, at least 42 *Haemaphysalis* spp. are indigenous to this region, representing almost half of its ixodid fauna [5]. In other words, 25% of the species in the latter genus occur in this area [5]. Therefore, Southeast Asia is regarded as the major evolutionary center for this genus [79].

Traditionally, the genus *Haemaphysalis* is subdivided into subgenera. Within the subgenus *Kaiseriana*, several species occur in Vietnam (Fig. 4) and in neighboring China [80]. Among these, *H. shimoga* was reported to occur in Vietnam (Table 1) and in Thailand [81]. On the other hand, its sibling species, *H. cornigera*, was not found to occur in the latter country but has been reported recently from Hainan Island of China, close to Vietnam [78]. Results of the present study attest that this species should also be added to the fauna of Vietnam. Adults of *H. cornigera* typically occur on carnivores, Cervidae and Bovidae [11], the latter also confirmed here.

Last but not least, *A. integrum* was identified here, for the first time, not only in Vietnam but also in the whole Indochinese subregion of Oriental Asia, considering that this species has been hitherto regarded as indigenous to only India and Sri Lanka [82]. In the present study, *A. integrum* was found on cattle which, together with buffalos, are typical hosts for this ixodid species [82], but it is also a frequent parasite of humans [83]. Based on the *cox*1 phylogenetic analysis in this study (Fig. 4), it is a sister species of the morphologically similar *A. geoemydae*. However, these two species might have been confused in the past, as reflected by (i) the contradicting mitochondrial marker sequences of identical isolates of *A. geoemydae* (e.g. SGL03d) in GenBank: high level of *cox*1 sequence identity (low p-distance) between a sequence of *A. geoemydae* deposited in GenBank (OL629478) and of *A. integrum* reported here whereas a very low level of 16S rRNA gene identity (high p-distance) between the corresponding sequence (OL616095) and of *A. integrum* reported here; (ii) ambiguous sequences reported under the name of *A. geoemydae* (e.g. KT382868 designated as *A. geoemydae* but having 100% sequence identity to the haplotype morphologically identified as *A. integrum* in this study). Interestingly, morphologically unidentified haplotypes that, based on the present study (Figs. 4, 5) belong to the phylogenetic clade of *A. integrum*, were reported from Myanmar as *Amblyomma* sp. [84]. Therefore, it is reasonable to suppose that *A. integrum* most likely occurs in a much broader geographical range that surrounds Vietnam, including probably the whole of Southeastern Asia.

## Conclusions

In this study, three tick species (*R. linnaei*, *A. integrum* and *H. cornigera*) are reported or were identified to the species level for the first time in Vietnam. Considering that none of their hosts were imported into the country, these findings increase the number of indigenous tick species to 62. Clade A of *R. microplus* and finally *R. linnaei* from the group of *R. sanguineus *s. l. occur in the country. There is multiple phylogenetic evidence that different species might exist among ticks reported under the name *R. haemaphysaloides* in South and East Asia. To our knowledge, this is also the first report of *A. integrum* in all of Southeast Asia, where this tick species almost certainly has a broad geographical distribution.

### Supplementary Information

Supplementary Material 1.

## Data Availability

The sequences obtained during this study are deposited in GenBank under the following accession numbers. *Cox*1 gene: PP197235-PP197241, 16S rRNA gene: PP197249-PP197254. All other relevant data are included in the manuscript and the supplementary material or are available upon request by the corresponding author.
