# Supplementary material for: A morpho-phylogenetic update on ixodid ticks infesting cattle and buffalos in Vietnam, with three new species to the fauna and a checklist of all species indigenous to the country
Source: Parasit Vectors. 2024 Jul 25;17:319. doi: 10.1186/s13071-024-06384-5 (PMC11282669; doi:10.1186/s13071-024-06384-5)
Supplement: Supplementary file 1 — Supplementary Material 1. [file 13071_2024_6384_MOESM1_ESM.pdf]

Supplementary Table 1. Data of tick collection from cattle, buffalo and dog.

Tube No.	Commune	Coordinates	District	Province	Host	Date	*Rhipicephalus microplus*				*Rhipicephalus linnaei*				*Amblyomma integrum*			
							L	N	M	F	L	N	M	F	L	N	M	F
1	Song Khua	20°55'55.3"N 104°52'30.0"E	Van Ho	Son La	Cattle	2023.01.10				24								
2	Song Khua	20°55'55.3"N 104°52'30.0"E	Van Ho	Son La	Cattle	2023.01.10		5	10	16								
3	Song Khua	20°55'55.3"N 104°52'30.0"E	Van Ho	Son La	Cattle	2023.01.10			3	3								
4	Nam Man	21°04'55.9"N 103°33'59.5"E	Song Ma	Son La	Cattle	2023.04.20												1
5 (NB 40 -2)	Nam Man	21°04'55.9"N 103°33'59.5"E	Song Ma	Son La	Cattle	2023.04.20								1				
6	Nam Man	21°04'55.9"N 103°33'59.5"E	Song Ma	Son La	Cattle	2023.04.20								1				
7	Song Khua	20°55'55.3"N 104°52'30.0"E	Van Ho	Son La	Cattle	2023.01.10			1	1								
8.1	Song Khua	20°55'55.3"N 104°52'30.0"E	Van Ho	Son La	Cattle	2023.01.10				5								
8.2	Song Khua	20°55'55.3"N 104°52'30.0"E	Van Ho	Son La	Cattle	2023.01.10				5								
9	Nguyen Khe	21°10'37.8"N 105°50'21.6"E	Dong Anh	Ha Noi	Cattle	2023.04.13	1	25	9	30								
10	Bac Hong	21°10'42.1"N 105°48'19.3"E	Dong Anh	Ha Noi	Cattle	2023.04.14		12	10	20								
11	Yen My	20°56'31.3"N 105°52'14.4"E	Thanh Tri	Ha Noi	Cattle	2023.04.16	1		1	70								
11.2	Yen My	20°56'31.3"N 105°52'14.4"E	Thanh Tri	Ha Noi	Cattle	2023.04.16				54								
12	Yen My	20°56'31.3"N 105°52'14.4"E	Thanh Tri	Ha Noi	Cattle	2023.04.16		15	91	10								
13	Yen My	20°56'31.3"N 105°52'14.4"E	Thanh Tri	Ha Noi	Cattle	2023.04.16		7	20	105								
14	Yen My	20°56'31.3"N 105°52'14.4"E	Thanh Tri	Ha Noi	Cattle	2023.04.16	1			2								
15	Yen My	20°56'31.3"N 105°52'14.4"E	Thanh Tri	Ha Noi	Cattle	2023.04.16				29								
16	Yen My	20°56'31.3"N 105°52'14.4"E	Thanh Tri	Ha Noi	Cattle	2023.04.16		2	5									
17	Yen My	20°56'31.3"N 105°52'14.4"E	Thanh Tri	Ha Noi	Cattle	2023.04.16		3	5	35								
17.2	Yen My	20°56'31.3"N 105°52'14.4"E	Thanh Tri	Ha Noi	Cattle	2023.04.16				15								
18	Yen My	20°56'31.3"N 105°52'14.4"E	Thanh Tri	Ha Noi	Cattle	2023.04.16		1	7	5								
19.1	Yen My	20°56'31.3"N 105°52'14.4"E	Thanh Tri	Ha Noi	Cattle	2023.04.16				8								
19.2	Yen My	20°56'31.3"N 105°52'14.4"E	Thanh Tri	Ha Noi	Cattle	2023.04.16				6								
19.3	Yen My	20°56'31.3"N 105°52'14.4"E	Thanh Tri	Ha Noi	Cattle	2023.04.16				18								
19.4	Yen My	20°56'31.3"N 105°52'14.4"E	Thanh Tri	Ha Noi	Cattle	2023.04.16		1	2	22								
19.5	Yen My	20°56'31.3"N 105°52'14.4"E	Thanh Tri	Ha Noi	Cattle	2023.04.16				8								
20.1	Bac Hong	21°10'42.1"N 105°48'19.3"E	Dong Anh	Ha Noi	Cattle	2023.04.14			1	12								
21.1	Yen My	20°56'31.3"N 105°52'14.4"E	Thanh Tri	Ha Noi	Cattle	2023.04.16				10								
21.2	Yen My	20°56'31.3"N 105°52'14.4"E	Thanh Tri	Ha Noi	Cattle	2023.04.16				11								
21.3	Yen My	20°56'31.3"N 105°52'14.4"E	Thanh Tri	Ha Noi	Cattle	2023.04.16				13								
21.4	Yen My	20°56'31.3"N 105°52'14.4"E	Thanh Tri	Ha Noi	Cattle	2023.04.16				11								
21.5	Yen My	20°56'31.3"N 105°52'14.4"E	Thanh Tri	Ha Noi	Cattle	2023.04.16	1	6	35	54								
21.6	Yen My	20°56'31.3"N 105°52'14.4"E	Thanh Tri	Ha Noi	Cattle	2023.04.16				12								
22.1	Song Khua	20°55'55.3"N 104°52'30.0"E	Van Ho	Son La	Cattle	2023.01.10				11								
22.2	Song Khua	20°55'55.3"N 104°52'30.0"E	Van Ho	Son La	Cattle	2023.01.10			1	17								
22.3	Song Khua	20°55'55.3"N 104°52'30.0"E	Van Ho	Son La	Cattle	2023.01.10				14								
23	Binh Long	21°39'40.9"N 106°11'34.9"E	Vo Nhai	Thai Nguyen	Cattle	2022.12.26				11								
24	Lien Hoa	20°59'44.6"N 104°51'30.7"E	Van Ho	Son La	Cattle	2023.01.11				7								
25	Binh Long	21°39'40.9"N 106°11'34.9"E	Vo Nhai	Thai Nguyen	Cattle	2022.12.26			9	13								
26	Thuy Lam	21°10'39.9"N 105°53'23.2"E	Dong Anh	Ha Noi	Cattle	2022.12.21				9								
27	Binh Long	21°39'40.9"N 106°11'34.9"E	Vo Nhai	Thai Nguyen	Cattle	2022.12.26				12								
28	Lien Hoa	20°59'44.6"N 104°51'30.7"E	Van Ho	Son La	Cattle	2023.01.11				1								
29	Hop Thanh	21°45'30.5"N 105°39'22.4"E	Phu Luong	Thai Nguyen	Catlle	2023.03.29	3	245	2			1						
29.2	Hop Thanh	21°45'30.5"N 105°39'22.4"E	Phu Luong	Thai Nguyen	Catlle	2023.03.29				7								
30.1	Yen Lang	21°41'32.0"N 105°30'12.0"E	Dai Tu	Thai Nguyen	Buffalo	2023.04.01				23								
30.2	Yen Lang	21°41'32.0"N 105°30'12.0"E	Dai Tu	Thai Nguyen	Buffalo	2023.04.01				15								
30.3	Yen Lang	21°41'32.0"N 105°30'12.0"E	Dai Tu	Thai Nguyen	Buffalo	2023.04.01				18								
30.4	Yen Lang	21°41'32.0"N 105°30'12.0"E	Dai Tu	Thai Nguyen	Buffalo	2023.04.01				17								
31	Nguyen Khe	21°10'37.8"N 105°50'21.6"E	Dong Anh	Ha Noi	Buffalo	2023.04.13				2								
57	Nguyen Khe	21°10'37.8"N 105°50'21.6"E	Dong Anh	Ha Noi	Cattle	2023.04.13	1	9	13	6								

Tube No.	Commune	Coordinates	District	Province	Host	Date	*Rhipicephalus microplus*				*Rhipicephalus linnaei*				*Haemaphysalis cornigera*			
							L	N	M	F	L	N	M	F	L	N	M	F
11	Tan Linh	21°07'28.8"N 105°23'15.5"E	Ba Vi	Ha Noi	Cattle	2022.09.21				3								
12	Tan Linh	21°04'40.1"N 105°25'31.3"E	Ba Vi	Ha Noi	Cattle	2022.09.22			1	3								
13	Tan Linh	21°12'40.5"N 105°27'23.5"E	Ba Vi	Ha Noi	Cattle	2022.07.02				2								
14	Tan Linh	21°12'40.5"N 105°27'23.5"E	Ba Vi	Ha Noi	Cattle	2022.07.02				2								
15	Muong Hung	20°56'39.8"N 103°52'33.9"E	Song Ma	Son La	Cattle	2022.07.07				33								2
16	Tan Linh	21°12'40.5"N 105°27'23.5"E	Ba Vi	Ha Noi	Cattle	2022.07.02		2	12	68								
17	Minh Chau	21°12'40.5"N 105°27'23.5"E	Ba Vi	Ha Noi	Cattle	2022.07.02				4								
18	Minh Chau	21°12'40.5"N 105°27'23.5"E	Ba Vi	Ha Noi	Cattle	2022.07.02				3								
19	Van Hoa	21°12'40.5"N 105°27'23.5"E	Ba Vi	Ha Noi	Cattle	2022.07.02		5	4	9			1					
20	Van Hoa	21°12'40.5"N 105°27'23.5"E	Ba Vi	Ha Noi	Cattle	2022.07.02				7								
21	Dong Thinh	21°52'35.0"N 105°35'48.0"E	Dinh Hoa	Thai Nguyen	Dog	2022.07.13						3	43	25				
26	Yen My	20°56'31.3"N 105°52'14.4"E	Thanh Trì	Ha Noi	Cattle	2022.07.02				36								
27	Linh Thong	22°01'07.0"N 105°40'00.0"E	Dinh Hoa	Thai Nguyen	Cattle	2022.07.13	2	3	34	114								
28	Minh Chau	21°12'40.5"N 105°27'23.5"E	Ba Vi	Ha Noi	Cattle	2022.07.02				1								
